# A comprehensive review of the progress of cell migration inducing hyaluronidase 1

**DOI:** 10.1097/MD.0000000000031610

**Published:** 2022-11-25

**Authors:** Xiangguang Miao, Yukai Wang, Zhiguo Miao, Haili Pan

**Affiliations:** a Neurological Institute of Jiangxi Province and Department of Neurology, Jiangxi Provincial People’s Hospital, The First Affiliated Hospital of Nanchang Medical College, Nanchang, Jiangxi, China; b Zhengzhou Traditional Chinese Medicine Hospital, Zhengzhou, China; c School of Chemistry and Chemical Engineering, Nanchang University, Nanchang, China; d Queen Mary School, Nanchang University, Nanchang, China.

**Keywords:** CEMIP (cell migration-inducing hyaluronidase 1), hyaluronidase 1, KIAA1199, tumor, migration, tumorigenesis

## Abstract

The gene cell migration inducing hyaluronidase 1 (CEMIP) is on chromosome 15q25 and codes for a 150-kDa protein with an N-terminal secretion signal, a G8 domain, 2 GG domains, and several repeats. It was first described as a specific protein in the inner ear relating to nonsyndromic hearing loss. Recently, increasing research detected its association in various cancers, determining the progression, metastasis, and prognosis by influencing the proliferation and invasion of the cells. This relation is accomplished through various interacting pathways, such as the Wnt/β-catenin signaling pathway and the epidermal growth factor receptor signaling pathway. Thus, CEMIP could be a novel and potential focus for tumor diagnosis and treatment, but further studies on the regulatory role of CEMIP in vivo and in vitro are still needed. Herein, we summarize the process in recent studies of CEMIP, especially in cancer research.

## 1. Introduction

**Figure 1.**
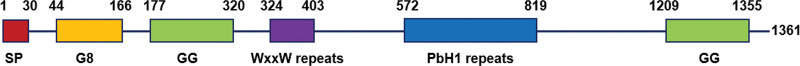
Schematic representation of the CEMIP secondary structure. SP: Signal peptide. G8 domain: G8 is known for the 8 conserved glycines, and it is predicted to contain an alpha helix and ten beta strands. GG domain: this domain is potentially relevant to CEMIP inactivation, including a beta-beta-alpha fold. It has been identified in members of the eukaryotic FAM3 family members and related proteins with unclear functions. WxxW repeats: this domain with unknown function carries a highly conserved WxxW sequence motif and has no less than 6 conserved cysteine residues. PbH1 repeats: Four parallel beta-helix repeats in the CEMIP. Proteins containing the repeats most often are enzymes with polysaccharide substrates. However, the function of the CEMIP PbH1 domain remains elusive. CEMIP = cell migration inducing hyaluronidase 1.

Cell migration inducing hyaluronidase 1 (CEMIP), also known as KIAA1199, was recruited in the Human Unidentified Gene-Encoded (HUGE) database as one of 1087 long cDNAs. KIAA family encodes large proteins with an estimated average length of 872 amino acids.^[1]^ There are several aliases for KIAA1199, such as colon cancer secreted protein 1 (CCSP1), hyaluronan-binding protein involved in hyaluronan depolymerization (HYBID), and transmembrane protein 2-like (TMEM2L). Currently, KIAA1199 is officially named CEMIP. The gene is situated on chromosome 15q25.1, which also has a brain tumor suppressor gene.^[2–4]^ CEMIP was initially reported as a specific protein in the nucleus and cytoplasm of the inner ear.^[5]^ It is a 150-kDa protein with a G8 domain in the N-terminal, which contains 5 duplicate β chain pairs and 8 glycine residues (Fig. 1). G8 domain is a potential transmembrane structure that is predicted to have signal peptides ^[6]^. It had been reported that mutations in the coding region of CEMIP could cause the development of nonsyndromic hearing loss.^[7]^

According to recently published studies, in terms of structure, the CEMIP protein has 3 other domains, with a secretion signal at the N terminal. CEMIP has 2 GG structural domains, each consisting of 7 β-strands and 2 α-helices with approximately 100 amino acid residues (Fig. 1). According to the phylogenetic tree, these 2 GG domains are derived from separate combinatorial incidents rather than intragene duplication. Additionally, the N-terminal of the 2 GG domains is more homologous to the *Dictyostelium* protein and the phage gp35 protein.^[8]^ Most CEMIP domains have undefined functions, such as GG domain, WxxW repeats, and PbH1 repeats (Fig. 1). Most G8-containing proteins with transmembrane segments or signal portions are positioned intra- or transmembrane.^[8,9]^ However, the mature CEMIP is verified to be localized in the cytoplasm instead of cellular membranes. Subcellular localization of CEMIP is limited to the perinuclear space, the ER, and then secreted outside.^[10]^

CEMIP expression is tightly regulated by genetic and epigenetic regulatory systems.^[11]^ Two parts around the CEMIP sequence (−1425 to −1135 and −125 to +27) are the enhancers of the gene in disease, specific to bind with NF-κB and AP-1, respectively. In contrast, the primary promoter activity of CEMIP depends on the DNA methylation status, with the active region located in the first intron region, a 1.9-kb long CpG island (−444 to +1509). This region in which methylation level is inversely proportional to the expression of CEMIP can be a potential therapeutic site. The less methylated the base sequence of the CpG island, the more advanced cancer, as evidenced by the demethylation of invasive breast cancer samples.^[11]^ Therefore, CEMIP regulates its gene expression through genetic and epigenetic mechanisms.

CEMIP can be secreted into the extracellular environment as an exosomal protein. Moreover, CEMIP promotes all 3 branches of the Wnt signaling pathways by modulating Ca^2 + ^signaling via interacting with the cell-membrane receptor Ephrin A2, WW domain binding protein 11 (WBP11), Protein tyrosine phosphatase type IVA member 3 (PTP4A3) or ER receptor inositol 1,4,5-trisphosphate receptor type 2 (ITPR2).^[12,13]^ Furthermore, the survival rate of solid cytoplasmic expression of CEMIP is one-quarter lower than that of nucleus strong expression at 4 years postoperatively.^[5]^ The nuclear confinement status of CEMIP illustrates the relative safety of cancer patients. It can also be used for early medical intervention or diagnostic indicators of tumor invasion.^[14,15]^

Although CEMIP expression in the nucleus has been found in colorectal adenomas, nuclear localization and overexpression of CEMIP may be associated with the presence of β-catenin regardless of the International Union Against Cancer stage.^[5]^ Comparing CEMIP-containing colorectal cancer (CRC) cells and their knockouts suggests that CEMIP is belonged to the Wnt signaling pathway, as changes in 8 Wnt axis genes, including β-catenin expression, share the same regulatory trends.

In this review, we summarize the cellular signaling pathways that CEMIP involves, describe the role of CEMIP in different cancers and hereditary hearing loss, and discuss the prospects of CEMIP research.

### 1.1. General properties of CEMIP

It has been reported that CEMIP is involved in several pathways. In the nearly 20 years since the first article on CEMIP and human disease was published, various possible interaction pathways for CEMIP have been reported.

## 2. CEMIP/PP2A/stathmin pathway

**Figure 2.**
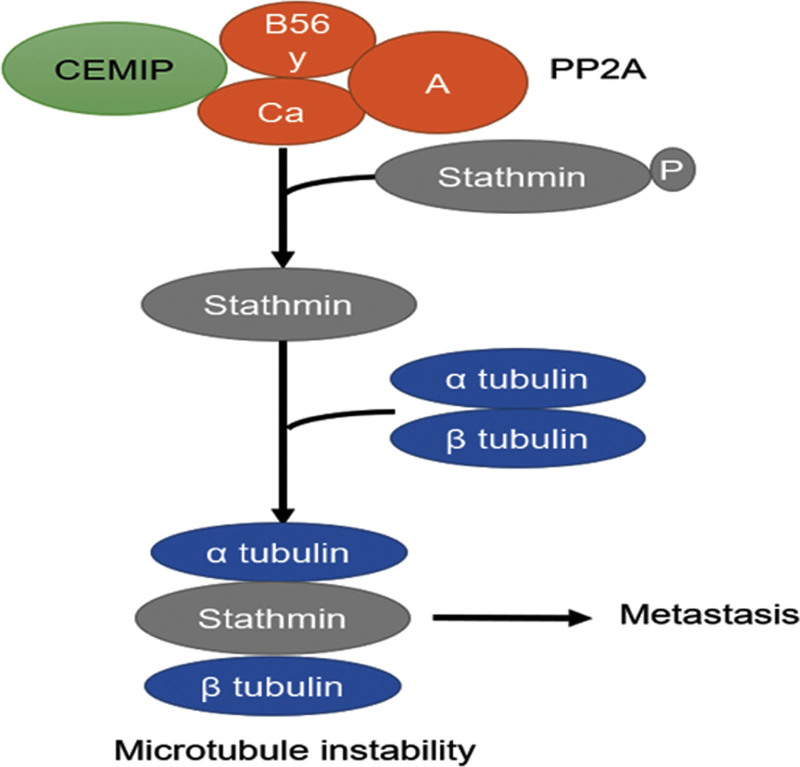
The proposed model of CEMIP-induced microtubule instability enhanced tumor metastasis. CEMIP interacts with PP2A (protein phosphatase 2A) and enhances its phosphatase ability, catalyzing dephosphorylation of stathmin. Unphosphorylated stathmin disturbs the microtubule formation, thus resulting in microtubule instability and, consequently, tumor metastasis. CEMIP = cell migration inducing hyaluronidase 1.

Protein phosphatase 2A (PP2A) belongs to serine/threonine phosphatases. PP2A is essential in several cellular signaling pathways, primarily regulating cell migration and survival.^[16]^ Based on co-immunoprecipitation and liquid chromatograph-mass spectrometer peptide sequencing of HCT116 cells, PP2A Cα and B56γ are demonstrated as the putative proteins interacting with CEMIP. Consequent experiments testified that a stable complex could be formed by CEMIP, PP2A Cα, and PP2A B56γ. The formed complex can induce PP2A Cα methylation, which is essential for the enzymatic activation of the PP2A holoenzyme, thus enhancing the phosphatase activity of PP2A. The complex causes the dephosphorylation of the stathmin, a major microtubule-destabilizing phosphoprotein.^[17]^ Unphosphorylated stathmin binds to and sequesters tubulin dimers to decrease the microtubule polymer, indirectly leading to microtubule instability.^[18]^ Microtubules regulate cell migration by driving forces as an integral part of the cytoskeleton.^[19]^ It has been reported that CEMIP-mediated destabilization of microtubules is essential to enhance the motility, invasion, and metastasis of tumor cells (Fig. 2).^[20,21]^

## 3. AMPK/GSK3β/β-catenin cascade

**Figure 3.**
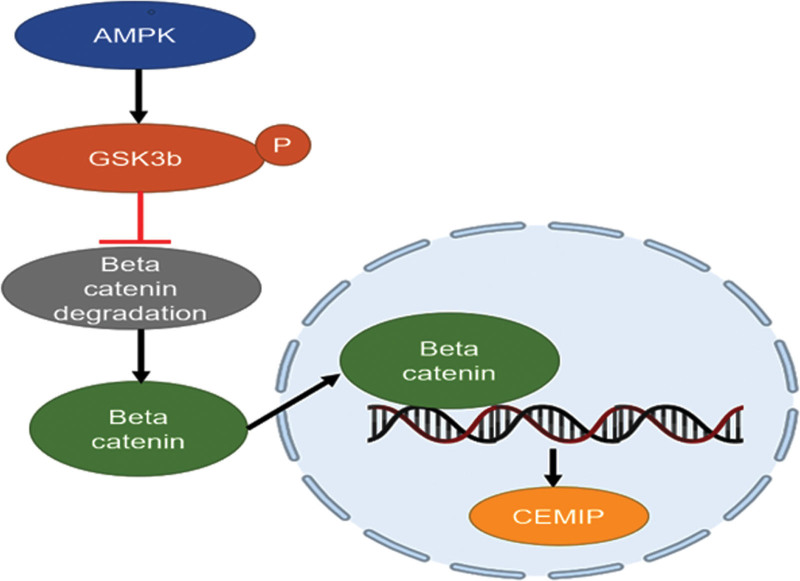
Proposed model of AMPK/GSK3β/β-catenin cascade with CEMIP. AMPK (AMP-activated protein kinase) promotes the phosphorylation of GSK3β (glycogen synthase kinase 3β), thus inhibiting its β-catenin degradation ability. β-Catenin can then enter the nucleus as a transcription factor, enhancing the expression of CEMIP. CEMIP = cell migration inducing hyaluronidase 1.

AMP-activated protein kinase (AMPK) has been identified as a cellular energy sensor, which is sensitive to and activated by energy stress. Once activated, AMPK inhibits acetyl-CoA carboxylases, which are responsible for the initial reaction that catalyzes the biosynthesis of fatty acids in all cells. Thus, the consumption of reduced nicotinamide adenine dinucleotide phosphate (NADPH) in fatty acid synthesis is reduced, while the product of NADPH by fueling fatty acid oxidation is elevated. This progress substantially decreases the generation of reactive oxygen species following extracellular matrix (ECM) stripping, averting anoikis and promoting cancer cell survival and migration.^[22]^ Elevated reactive oxygen species production leads to glycogen synthase kinase 3β (GSK3β) phosphorylation, thus inhibiting its activation. The GSK3β is the regulator of β-catenin, and phosphorylated (p)-GSK3β can increase β-catenin activity by enhancing its stability. Therefore by contributing to reduced degradation, elevated intracellular concentration, and nuclear cumulation of β-catenin, GSK3β/β-catenin is crucial in the modulation of several genes regulating cellular growth, differentiation, and metastasis.^[23]^ This impact triggers the downstream transcription of specific target genes, enhancing cell survival growth and metastasis..^[24]^ CEMIP is a putative target gene, and its overexpression appears when the AMPK/GSK3β/β-catenin cascade is activated (Fig. 3). Such overexpression of CEMIP is detected in colon cancer tissues, indicating that CEMIP may be involved in the progression of local tumors.^[25]^

## 4. NF-κb signaling pathway

Nuclear factor-κB (NF-κB)-activating cascades play a prominent role in inflammation and immunity response. When aberrantly activated, they increase the expression of proto-oncogenes, anti-apoptotic genes, and cell cycle proteins, promoting cancer metastasis^[26,27].^ In the NF-κB cascades, NF-κB p50, NF-κB p65, and B-cell lymphoma-3 (BCL-3) are responsible for the carcinogenic potential.^[28]^ Based on the study on the transformed keratinocytes with infinite reproduction ability, both BCL-3 and NF-κB p65 promote CEMIP expression levels.^[29]^

## 5. CEMIP as a linkage between semaphorin 3a/plexin 2a and EGFR signaling

**Figure 4.**
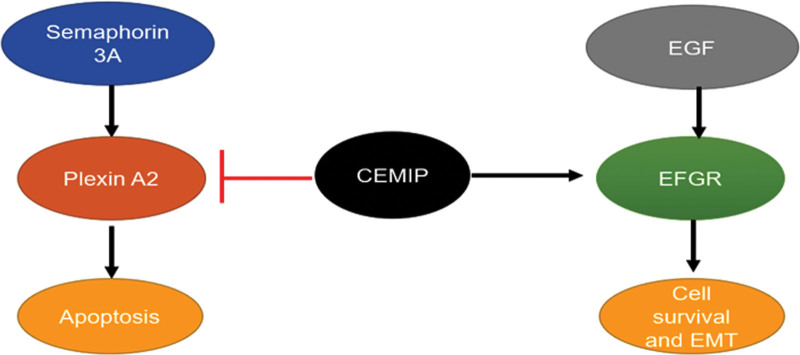
The proposed model of Semaphorin 3A/Plexin 2A and EGFR (epidermal growth factor receptor) signaling is linked by CEMIP. Semaphorin triggers apoptosis in a plexin A2-dependent manner. At the same time, EGF promotes cell survival and EMT (epithelial–mesenchymal transition) by binding to EGFR. CEMIP serves as a bridge between these 2 pathways by inhibiting plexin A2 and supporting EGFR. CEMIP = cell migration inducing hyaluronidase 1.

Plexin A2 was identified to bind with the G8 domain of CEMIP-through yeast 2-hybrid experiments.^[18,29]^ Typically, Plexin A2 is responsible for class 3 signaling semaphorin bound to Neurospherins, and sustained activation of class 3 signaling Semaphorin can lead to apoptotic cell death.^[30,31]^ Mechanistic investigations revealed that CEMIP blocks the semaphorin 3A- and plexin A2-dependent apoptotic pathway in cervical cancer cells as they trigger apoptosis only on CEMIP deficiency. How CEMIP blocks the apoptosis may depend on promoting the stability and signaling of epidermal growth factor receptor (EGFR), and the result supports that cell death rises even more dramatically in response to semaphorin 3A stimulation in EGFR-deficient cells. In addition, it suggests that semaphorin 3A can drive the apoptotic pathway when the activity of EGFR is inhibited.^[29]^ Moreover, as an EGFR-binding protein, CEMIP can promote phosphorylations of EGFR, sarcoma gene, and mitogen-activated protein kinase 1 (MEK1), indicating that CEMIP links EGFR to downstream kinases. Of note, as epidermal growth factor (EGF) can trigger epithelial-mesenchymal transition (EMT) in CaSki cells, CEMIP may relate to the EMT (Fig. 4). Results of immunofluorescence analysis demonstrate that deficiency of CEMIP disturbs the re-localization of E-cadherin and zona occludens 1(ZO1) in CaSki cells dependent on EGF-mediated stimulation.^[29]^ Therefore, CEMIP promotes EGF-induced EMT and relates to cell invasion in cervical cancer cells.

## 6. Wnt/β-catenin signaling pathway

The Wnt signaling pathway, as a multichannel signaling pathway, is initiated by the binding between ligand Wnt protein and related receptors in the membrane. Wnt proteins primarily bind to Frizzled (Fzd) and low-density lipoprotein receptor-related protein 5/6 (LRP5/6), and both are cell surface receptors, thereby inhibiting GSK-3β and casein kinase 1 (CK1). This repression facilitates the stability of cytoplasmic β-catenin and the ultimate translocation of β-catenin to the nucleus. The interaction with the T-cell factor/lymphoid enhancer factor (TCF/LEF) happens, and the specific gene is activated.^[32]^ The results of chromatin immunoprecipitation analysis showed that 4 binding regions surround the CEMIP locus for TCF4. Since TCF4 protein is a critical transcription factor in the Wnt/β-catenin signaling, CEMIP has been deemed a target gene for this pathway.^[33,34]^ As a core component of this pathway, β-catenin plays a prominent role in regulating multiple genes that participate in cell proliferation, differentiation, and invasion.^[34]^ CEMIP and Wnt are both highly expressed in colorectal adenomas and carcinomas, indicating that CEMIP is a positively regulated protein in this pathway and a presumed marker of transformation in colorectal adenomas.^[35]^

**Figure 5.**
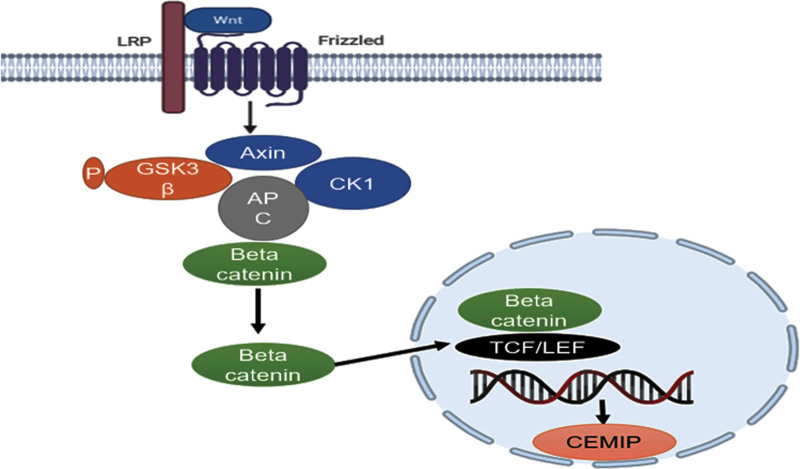
Proposed model of CEMIP in Wnt/β-catenin pathway. When the classical Wnt signaling pathway is turned on, the destruction complex formed by Axin, CK1 (casein kinase 1), APC (APC regulator of Wnt signaling pathway), and GSK3β (glycogen synthase kinase 3b) is inactivated to stabilize the β-catenin. Thus, the β-catenin can be transported to the nucleus for modulating transcription of CEMIP. CEMIP = cell migration inducing hyaluronidase 1.

Moreover, according to an experiment in NCI-N87 and AGS cells, knockdown of CEMIP decreases β-catenin expression. Thus, in the cytoplasm, the accumulation of β-catenin is disturbed, causing less β-catenin to enter the nucleus. This process suppresses the initiation of the Wnt/β-catenin signaling pathway. Afterward, both decreased expression of c-Myc and cyclin D1, 2 major downstream proteins involved in this pathway, is detected. Based on these results, it is proposed that CEMIP correlates with cancer invasion and metastasis ability through the Wnt/β-catenin signaling pathway (Fig. 5).^[36]^

### 6.1. CEMIP and tumorigenesis

CEMIP has been associated with several human diseases or conditions characterized by abnormal cell migration and proliferation (Table 1). Based on the studies, several kinds of tumors are involved. However, although CEMIP upregulation is associated with promoting tumors in most cancers, CEMIP may be antineoplastic in specific cancers.

**Table 1:** The function of CEMIP in different cancers.

Cancer	Function		References
	In vivo	In vitro	
Colorectal cancer	Diagnosis, medical intervention, and prognosis	Cell proliferation, invasion, and migration	^[5, 11, 14, 19, 25, 32, 35, 37,39-42, 61]^
Breast cancer	Diagnosis, medical intervention, and prognosis	Cell migration, proliferation, apoptosis, and metabolism	^[11, 15, 32,41-45]^
Pancreatic cancer	Diagnosis and prognosis	Cell migration and invasion	^[46-49]^
Gastric cancer	Diagnosis	Cell proliferation and migration	^[38-62]^
Lung cancer		Cell proliferation and migration	^[59]^
Chondrosarcoma	Diagnosis and tumorigenesis	Cell invasion and migration	^[60, 63]^
Prostate cancer	Tumorigenesis	Cell proliferation, migration, invasion, and apoptosis	^[20, 50-54]^
Ovarian cancer		Cell migration, invasion, and apoptosis	^[55]^

CEMIP = cell migration inducing hyaluronidase 1.

#### 6.1.1. Colorectal cancer.

Recent studies have shown that CEMIP upregulation is related to poor survival and neoplastic recurrence in CRC patients. Furthermore, CEMIP is identified as a potential target of miR-140-3p, promoting the proliferation and invasion of CRC cells and reverting miR-140-3p-induced anti-proliferative effects by modulating the expression of several epithelial marker proteins. Additionally, miR-140-3p expression is negatively associated with CEMIP and is an independent prospective factor for tumor regression for CRC patients.^[37]^ Several studies have demonstrated that down-regulating CEMIP by miR-216a can inhibit the invasion and metastasis of CRC cells, revealing the regulatory role of the miRNA-CEMIP axis in CRC.^[38]^ Individual studies have identified the relationship between the expression of CEMIP and EMT. Taurine-upregulated gene 1 (TUG1) promotes the expression of CEMIP and accelerates EMT and metastasis of CRC cells by inhibiting the expression of miR-600.^[39]^ Furthermore, in vitro experiments have shown that overexpression of cancer susceptibility 19 (CASC19) can enhance the proliferation and migration of CRC cells with the upregulation of CEMIP and EMT markers.^[40]^

#### 6.1.2. Breast cancer.

CEMIP expression is upregulated in invasive breast cancer specimens and negatively correlated to patient survival. CEMIP silencing in MDA-MB-435 cancer cells leads to the transformation of mesenchymal cells into epithelial cells, thereby reducing the ability of cells to migrate in vitro. Gain-of-function experiments provide further confirmation of the role of CEMIP in migration. Enhanced cell migration by CEMIP requires ER localization, which binds to its partner binding immunoglobulin (BiP) to form a stable complex. A novel ER-retention motif is found in CEMIP, which is necessary for ER localization and BiP interaction. It is found that CEMIP mediates ER calcium leakage, resulting in an accumulation of cytoplasmic calcium, thus leading to activation of protein kinase C (PKC) and, ultimately, cell migration.^[41]^ MDA-MB-231 and HS578T cell lines decrease cell motility and proliferation ability after the CEMIP gene is knocked out. In addition, quantitative proteomics analysis shows that CEMIP knockout in MDA-MB-231 affects many cell functions, including apoptosis, metabolism, and cell motility.^[42]^ In general, tumor cells are in a strict microenvironment because of their accelerated and uncontrolled growth rate and demands for high nutritional supplementation. Individual studies have provided insights into the link between CEMIP and BiP expression and their role in promoting survival in hypoxia. BiP signaling downstream of CEMIP regulates cell resistance to hypoxia. Decreasing BiP in cells via regulating CEMIP expression makes the cells sensitive to hypoxia therapy, reduces glucose uptake, and leads to tumor regression in vivo.^[43]^

#### 6.1.3. Pancreatic cancer.

In previous studies, CEMIP has been investigated and reported to be correlated to early detection, cancer cell migration, invasion, and poor prognosis.^[44–46]^ Suh et al proposed that CEMIP is probably valuable for the early detection of pancreatic cancer, while Koga et al showed that it is associated with cancer prognosis.^[44]^ Increased expression of CEMIP may partially promote the aggressive phenotype by increasing the concentration of low molecular weight hyaluronan. A possible link between inflammation and enhanced migration is induced by CEMIP in pancreatic ductal adenocarcinoma.^[45]^ However, the small sample size makes it difficult to draw a reliable conclusion based on these previous experiments. Recently, a novel point has explored the relationship between CEMIP and pancreatic cancer. CEMIP can be a potential marker for pancreatic cancer. The integrated measurement of serum cancer antigen 19-9 (CA19-9) and CEMIP concentration could be a novel laboratory method for diagnosing pancreatic cancer clinically.^[47]^

#### 6.1.4. Gastric cancer.

CEMIP is upregulated in gastric cancer tissues and correlates to poorer clinical results in gastric cancer. CEMIP promotes the invasion and migration of gastric cancer cells by enhancing the Wnt/β-catenin pathway and matrix metalloproteinases-mediated EMT progression.^[36]^

#### 6.1.5. Lung cancer.

In nonsmall cell lung cancer cells, reduced CEMIP suppresses proliferation and migration of nonsmall cell lung cancer cells and down-regulated the expression of several transcription factors related to the EMT process and EGFR signaling.^[48]^

#### 6.1.6. Chondrosarcoma.

Recently, individual studies have shown that increased expression of CEMIP contributes to antitumor activity. CEMIP expression suppresses cell invasion and migration in rat chondrosarcoma cells rather than affecting cell proliferation and apoptosis. Additionally, due to changes in the tumor microenvironment, such as inhibition of ECM formation, CEMIP expression prominently inhibits the growth of transplanted tumors and suppresses the staining ability of Achillean blue in tumor tissues.^[49]^ The antitumor effect of CEMIP provides a new idea for us to carry out new research.

#### 6.1.7. Prostate cancer.

CEMIP is reported to involve prostate cancer proliferation,^[50]^ migration, and invasion.^[51]^ It was found that CEMIP promotes anoikis resistance by enhancing protective autophagy^[52,53]^ and facilitates ferroptosis resistance by promoting cystine uptake^[54]^ in prostate cancer cells during ECM, thus promoting metastasis formation. These studies provide new insight into therapeutic strategy development for prostate cancer.

#### 6.1.8. Ovarian cancer.

CEMIP was found to play an essential role in ovarian cancer progression. CEMIP expression is significantly upregulated in ovarian cancer tissues. Ovarian cancer cells’ migration and invasion capacity are significantly decreased, and the proportion of apoptotic cells increases after silencing CEMIP.^[55]^

#### 6.1.9. Other cancers.

CEMIP is also reported to involve in other cancers, such as gallbladder cancer,^[56]^ endometrial cancer,^[57]^ and papillary thyroid cancer.^[58]^

### 6.2. CEMIP and hereditary hearing loss

Hereditary hearing loss is a highly genetically heterogeneous sensory disorder. According to the cDNA microarray analysis and semiquantitative RT-PCR experiments, CEMIP has predominantly expressed in Deiters’ cells and the spiral ligament of the inner ear, especially.^[34]^

Based on the screening results of the CEMIP gene for mutations in patients with nonsyndromic hearing loss in Japan, 3 possible point mutations related to this disease are selected: an Arg187-to-Cys (R187C) mutation, an Arg187-to-His (R187H) mutation, and a His783-to-Tyr (H783Y) mutation. The H783Y mutation exhibits an abnormal pattern of cytoplasmic distribution that may be the basis of the molecular mechanism of hearing impairment.

Further transiently transfection experiments demonstrated that the R187C and R187H mutations do not affect the subcellular localization of the gene product in vitro, while the H783Y mutation emerged in a worm-eaten pattern, suggesting the potential involvement of hearing impairment.^[7]^ However, based on the large-scale screening, in Japanese patients, the primary causes of hearing loss are mutations in gap junction protein beta 2 (GJB2), solute carrier family 26 member 4 (SLC26A4), cadherin-related 23 (CDH23), and mitochondrial DNA 1555A→G mutation. In independent autosomal dominant families, mutations of CEMIP and multiple related genes have been detected.^[59]^ Also, in various supporting cells in the organ of Corti, distribution of CEMIP is detected, but the specific function of CEMIP in these cells is still unclear.^[59]^

### 6.3. Prospects of CEMIP research

Previous results suggested that CEMIP might be a diagnostic marker for several cancers, such as cholangiocarcinoma (CCA), gastric cancer, and CRC.^[60–62]^ As a secreted protein, overexpression of CEMIP is detected in serum levels in CCA patients before curative surgery. Moreover, the attenuated serum CEMIP level may be a marker for estimated poor prognosis in patients with CCA. Based on clinical records, the serum CEMIP as a diagnostic marker is more accurate than the traditional diagnostic marker CA19-9, suggesting that CEMIP can serve as a novel cancer detection marker.^[60]^ However, much previous research relating to CEMIP and cancers only focused on verifying the potential role of CEMIP in diverse cell lines, and the overexpression of secreted CEMIP was always ignored. Additionally, as the potential antitumor ability in chondrosarcoma, the relation between serum level and cancer diagnosis and prognosis becomes slightly unclear. Further research in vitro needs to be performed to verify whether the serum level of CEMIP can serve as a standard marker in clinical medicine, facilitating the early phase diagnosis and prognosis.

In addition, human microRNA genes are detected frequently in cancer-related genomic regions, participating in the diverse biological and physiological processes involving proliferation and migration.^[63,64]^ The expression level of CEMIP is negatively associated with miR-216a, and this correlation is verified in CRC tumor tissues. The luciferase reporter assays confirm the association, indicating that CEMIP is the direct target of miR-216a. Also, another microRNA miR-188-5p is reported as the regulator in RA, suggesting that microRNA-KIAA199 interacting plays a prominent role in CEMIP-related diseases.^[5]^ However, the detailed mechanism of microRNA still awaits elucidation. Whether the microRNA interact with other binding proteins in the regulation procedure? What domain is the most selected binding site? Can specific microRNAs serve as therapeutic tools for CEMIP-related diseases such as cancer? These open questions need more research to be clarified.

## 7. Conclusion remarks

CEMIP is a prominent element in hearing loss, keratoconus with cataracts, rheumatic heart, osteoarthritis, and cancers as a multidomain protein involved in various interacting pathways. It is usually deemed as a protumor factor in the progression of cancers. Overexpression is related to metastasis and poor prognosis of cancers that several CEMIP and tumors research detected. However, another study indicated that CEMIP has antitumor characteristics, leading to a more complex role in cancer regulation. As a potential marker of cancers, CEMIP can associate with several types of microRNAs to be a novel therapeutic target for cancer treatment. Overall, CEMIP is a novel target for cancers and other human diseases such as CRC, CCA, and prostate cancer, further investigations on the regulation of CEMIP are awaited.

## Acknowledgments

We thank Shunqi Wang (Nanchang University) for his work on the English revision of the manuscript.

## Author contributions

**Data curation:** Xiangguang Miao, Yukai Wang.

**Formal analysis:** Zhiguo Miao.

**Project administration:** Xiangguang Miao.

**Resources:** Xiangguang Miao.

**Supervision:** Zhiguo Miao, Haili Pan.

**Writing—original draft:** Xiangguang Miao, Yukai Wang.

**Writing—review and editing:** Xiangguang Miao, Yukai Wang, Haili Pan.
